# Evaluation of Well Designs to Improve Access to Safe and Clean Water in Rural Tanzania

**DOI:** 10.3390/ijerph15010064

**Published:** 2018-01-04

**Authors:** Aminata Kilungo, Linda Powers, Nathan Arnold, Kelli Whelan, Kurt Paterson, Dale Young

**Affiliations:** 1 Mel and Enid Zuckerman College of Public Health, Health Promotion Sciences Department, The University of Arizona, 1295 N Martin Avenue, Tucson, AZ 85721, USA; 2 Electrical and Computer Engineering, Biomedical Engineering, The University of Arizona, 1230 E. Speedway Blvd, Tucson, AZ 85721, USA; lspowers@email.arizona.edu; 3 Department of Civil and Environmental Engineering, Michigan Technological University, 801 Dow Building, Houghton, MI 49931, USA; nbarnold@mtu.edu (N.A.); kmwhelan@mtu.edu (K.W.); 4 Department of Engineering, James Madison University, 801 Carrier Dr., Harrisonburg, VA 22807, USA; paterson@mtu.edu; 5 Maji Safi kwa Afya Bora Ifakara (MSABI), Kilosa Road 65, Morogoro 284, Tanzania; africadale@gmail.com

**Keywords:** borehole, coliform, *Escherichia coli*, groundwater, Ifakara, water quality, well design, well depth, well comparison, Sub-Saharan Africa, Tanzania

## Abstract

The objective of this study was to examine three well designs: drilled wells (20–30 m deep), closed dug wells (>5 m deep), and hand-dug open wells (<5 m deep), to determine the water quality for improving access to safe and clean water in rural communities. Heterotrophic plate count (HPC), total coliforms (TC), *Escherichia coli* (*E. coli)* and turbidity, were used to assess the water quality of 97 wells. Additionally, the study looked at the microflora diversity of the water, focusing on potential pathogens using outgrowth, PCR, and genome sequencing for 10 wells. Concentrations of TC for the open dug wells (4 × 10^4^ CFU/100 mL) were higher than the drilled (2 × 10^3^ CFU/100 mL) and closed dug wells (3 × 10^3^ CFU/100 mL). *E. coli* concentration for drilled and closed dug wells was <22 MPN (most probable number)/100 mL, but higher for open wells (>154 MPN/100 mL). The drilled well turbidity (11 NTU) was within the standard deviation of the closed well (28 NTU) compared to open dug wells (49 NTU). Drilled and closed wells had similar microbial diversity. There were no significant differences between drilled and closed dug wells. The covering and lining of hand-dug wells should be considered as an alternative to improve access to safe and clean water in rural communities.

## 1. Introduction

Sub-Saharan Africa is inhabited by a total of 783 million people, 40% of which do not have access to safe and clean water [1]. The region failed to meet its 2015 Millennium Development Goal (MDG) to improve access to safe and clean water for 40% to 75% of its population [1]. Tanzania is one of those countries that did not meet this goal. According to the World Bank, the percentage of rural Tanzania with improved access to safe and clean water was 45% in 1990 and 46% in 2015 [2]. It is important to note that while Tanzania did not make significant progress, other rural populations in the world have made progress to improve access to safe and clean water from 62% in 1990 to 85% within a similar time-frame [2]. As we move to the next phase of the Sustainable Development Goals (SDG), it is important to examine how interventions could be carried out differently to foster ownership and sustainability beyond donor funding. In this case, sustainability of these water sources would at least allow their continued function to provide safe and clean water to the community, which includes the ability to be repaired when broken or damaged. Solutions need to focus on cost-effective and feasible technologies to which the communities have access, and low-cost intervention techniques are needed to allow the rural community at large to invest and participate in a manner that is appropriate for their environment and limited resources. As recently pointed out by the International Institute for the Environment and Development (IIED), up to 360 million U.S. dollars have been spent to drill boreholes in Africa. Once these boreholes break down, the communities cannot afford to repair them, and they often fail within a few years [3]. Even though Community Based Management (CBM) of the water supply has not proven to be effective in most rural communities [4,5], reforming CBM and building upon existing community efforts may be one of the options for some communities to improve access to safe and clean water. 

**Figure 1:**
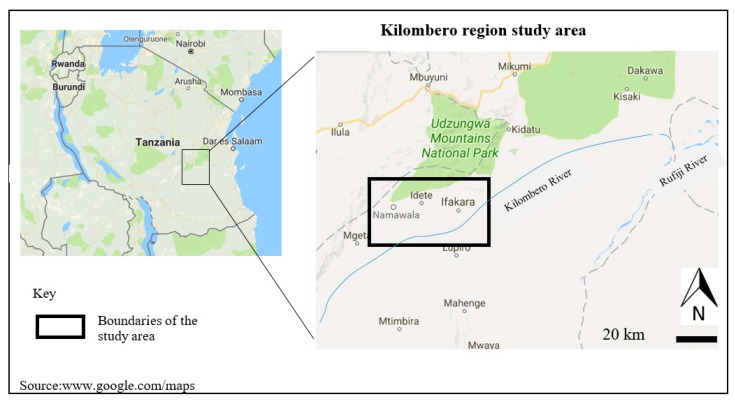
Map of the Kilombero region, Tanzania.

In 2010, a non-profit organization, Maji Safi kwa Afya Bora Ifakara (MSABI: clean water for better health in Ifakara), began a project to improve rural water sources in the Kilombero region by drilling boreholes 20–30 m deep. The study focused on three wards within the Kilombero region in Tanzania; Ifakara, Namawala and Idete (Figure 1). The region is approximately 13,545 km^2^ with a total population of 407,880 [6]. The population of Ifakara alone is approximately 55,956, while that of Idete is 21,648 [6]. Data for Namawala at the ward level (the lowest population count) is not included in the Tanzania National Statistics, but is estimated to be similar to that of Namawala. The Kilombero River has several tributaries and streams that serve as water sources for most of the villages in the three areas.

To improve access to safe and clean water, MSABI began installing drilled rope pumps. These are lined and covered boreholes. They also started another project to modify hand-dug open wells into closed wells by providing lining, installing a locally made rope pump and covering the top of the well. Prior to MSABI’s efforts, it was estimated that the region had over 750 water points with more than 75% being shallow dug wells and surface water [7]. Less than 25% of the population had access to safe and clean water and less than 15% had improved sanitation [7,8]. By 2011, MSABI had installed 150 drilled boreholes and had started a project to cover and line the hand-dug wells to protect them from any outside contamination. 

This study compared three types of water sources: deeper drilled boreholes (20–30 m), hand-dug wells converted into closed wells (depth varies but between 5 and 10 m), and the shallow hand-dug wells (depth < 5 m). The hand-dug wells with depth > 5 m are internally lined and the tops are covered. These two improved well designs (the borehole and the open top converted into a closed well) will be referred to as MSABI rope pump well, and closed well, respectively. Improved water sources as defined by the Joint Monitoring Programme (JMP) are sources that, by their very nature of construction, adequately protect the source from outside contamination in particular with fecal matter [9]. Examples of improved water sources include boreholes and protected dug wells [10]. The third design is an unimproved shallow well (<5 m deep) and will be referred to as an open well. The MSABI rope pump well averages 20–30 m in depth and 0.15 m in diameter. A concrete sanitary seal is applied to the soil clay layer between 4 and 10 m in depth and the remainder of the hole is lined by a polyvinyl chloride (PVC) pipe. The wellhead is protected by a plastered cement slab. Water enters at the bottom of the borehole through a slot covered by a filter screen, and the well is equipped with a rope pump to draw water to the surface. The rope pump uses a basic pulley and wheel system, with a rope and piston to pull water to the surface using a handle [7]. The MSABI rope well, including the rope pump, costs around US$1000 [7,11]. Other drilled boreholes cost anywhere between $8000 and $12,000 [11,12]. To offset high costs to the community, MSABI raised funds to cover a portion of the costs associated with the installation. The hand-dug wells converted to closed wells average >5 m in depth and are lined by a concrete plaster which starts from the bottom of the well upwards from 3 to 5 m. The plaster is applied to the locally made bricks (lean cement/sand mix 1:15) that line the well. The well liners and plastered cement slab protection on the wellhead prevents outside contamination and murky water from entering the well, especially during and after rainfall. This type of well is also equipped with the same rope pump as the MSABI rope pump, and uses the basic pulley and wheel system and a rope and piston to pull water to the surface. Since the designs vary, the costs also vary, however, it remains cheaper than the cost of installing a new borehole, and can be done locally without requiring drilling machines. The open wells in the region tend to be shallow with an average of 3 m in depth, some of which are lined with bricks. These wells are not equipped with any type of water pumping system. None of the wells had any types of fences or barriers to prevent livestock or animals from getting closer to the wellhead. 

The research question in this study was to examine the benefits of deeper, or shallow protected wells, and whether deeper wells offer water of better quality than shallow wells. The findings of this study will provide further guidance on access and water supply development in Tanzania and Sub-Saharan Africa as a whole. 

## 2. Materials and Methods

A total of 97 samples were collected with an average of 32 wells from each village: Ifakara, Namawala, and Idete. Approximately 11 of each of the three kinds of wells (MSABI rope pump wells, closed wells, and open wells) were sampled from each village. Due to limited resources, only 10 wells from Ifakara village were analyzed for microflora focused on potential pathogens. Three of each well design were analyzed. The types of wells and depths are included in Table 1. The resources that were available to facilitate water sample collection included the MSABI offices, who provided guidance on well identifications and well specifications and Ifakara Health Institute (IHI) which provided laboratory space. 

### 2.1. Water Microbial Quality Assessment

Water samples were collected using plastic sterile whirl-packs and transported to the Ifakara Health Institute (IHI) laboratory using an ice chest. The sampling took place from early morning until late afternoon and the water samples were processed within 8 h of collection. For heterotrophic plate counts and total coliforms, 1 mL of each sample was cultured onto a 3 M Heterotrophic Count Plate (AQHC) Petrifilm (3 M, St. Paul, MN, USA) via spread plating after dilution in phosphate-buffered saline (0.1, 0.01, and 0.001 mL dilutions). Heterotrophs, which require organic carbon for growth, were plate counted for well comparison purposes only. Coliforms are a group of bacteria which are aerobic and facultative anaerobic, Gram-negative, non-spore formers, rod-shaped, and ferment lactose with the formation of gas within 48 h at 35 °C. Total coliforms are naturally present in the environment. Even though total coliforms are not useful as indicators of fecal pathogens, they are, however, useful indicators of other pathogens in drinking water [13]. For undisinfected groundwater, TC (total coliforms) may indicate a pathway for surface or near-surface pathogen entry into the source water as well as providing a warning that soil microorganisms may have entered the well [14]. The number of *E. coli* was determined using Colilert Test Kits (IDEXX, Westbrook, ME, USA). *E. coli* is a fecal coliform, or bacteria, whose presence indicates fecal contamination from human or animal waste [14]. The test kit used a pre-dispensed substrate which was added to 10 mL volumes of the water samples. This method results in a most probable number (MPN). All samples were incubated at 35 °C for 24 h. The results were then multiplied by 10, to determine the MPN per 100 mL. We did not have enough of either of the two tests for coliform (i.e., 3 M Petrifilm or Colilert Test Kits), for all the wells. For this reason, we could only sample a limited number of the wells for *E. coli* assessment using the Colilert test. 

### 2.2. Water Microbial Diversity Assessment 

In addition to the detection of microbial water quality indicators, microflora diversity was also of interest for comparison purposes. However, due to limitations of the types of analysis that can be done in remote areas, water samples were collected and shipped to the United State via express delivery, which were then collected by a trained MSABI technician. The water samples were collected in 15 mL polypropylene sterile tubes, placed on ice in a cooler, and were processed immediately upon arrival four days after collection.

Different types of media were used to facilitate the growth of the various types of bacteria. Hemolytic bacteria were of interest in this study since they produce hemolysis which is associated with virulence [15] and exotoxins that lyse red blood cells which are common to infectious strains [16,17]. Blood agar is used to test for β-hemolysis after a complete lysis of the red blood cell which allows prediction of bacterial pathogens [18]. Since some samples were turbid, and interferences by environmental contaminants in the polymerase chain reaction [19] inhibit Taq polymerase, especially in the presence of organic and inorganic compounds [20], the samples were cultured to isolate bacterial colonies prior to performing PCR. To culture hemolytic bacteria, 0.1, 0.01, and 0.001 mL of each water sample was inoculated on blood agar plates (Tryptic Soy Agar with 5% sheep blood). The blood agar plates were incubated both aerobically and anaerobically at 35 °C for 1–4 days (or until colonies appeared), to increase the recovery of diverse hemolytic bacteria. To recover stressed and slow-growing bacteria, water samples of 0.1, 0.01 and 0.001 mL were inoculated on R2A agar plates and incubated at 35 °C for 5 days. To culture other rapidly growing bacteria, 0.1, 0.01, and 0.001 mL of each water sample was inoculated on Luria-Bertani (LB) agar plates, a nutrient-rich medium, with incubation at 35 °C for 24 h. To further increase microbial diversity, an enrichment growth technique was also carried out. One mL of water sample was inoculated in alkaline peptone water (BBL Alkaline Peptone Water), an enrichment medium (Becton, Dickinson and Company, Sparks, MD, USA), with incubation at 35 °C for 8 h, followed by inoculation onto Thiosulfate Citrate Bile Salts agar (TCBS) plates with incubation at 35 °C for 24 h. 

A total of 93 colonies were selected for further species identification tests. These included the β hemolytic colonies, totaling 50 colonies from the blood agar plates: all 12 colonies from the TCBS agar plates, 22 selected representative colonies from LB based on diversity, and nine colonies from R2A plates. Only one colony of the same color, morphology, surface, and opacity was selected from the R2A plates. The PCR DNA template was prepared using QIAquick PCR purification kits following the manufacturer’s protocol (QIAGEN, Valencia, CA, USA).

Universal V3 primers were used for both forward and reverse PCR. The V3 primers amplify the 16 s rRNA. These amplify DNA that codes for a ribosomal subunit, a highly conserved region found in all bacteria. They were selected because of their ability to provide sufficient phylogenic information on bacteria [21,22] as the V3 region tends to be longer and provides a better resolution than many other genes and can be unambiguously mapped to the genus level approximately 97% to 99% of the time [21]. The PCR was performed with the following cycle conditions: 95 °C (denaturation) for 30 s, 60 °C (annealing) for 1 min, and 72 °C (extension) for 45 s for a total of 40 cycles (BioRad MJ Mini Personal Thermocycler, Hercules, CA, USA). The PCR mix without any added DNA was included as a negative control. Amplicon sequencing was performed at the University of Arizona Genetic Core laboratory (Tucson, AZ, USA). Forward and reverse strands were aligned, and only the aligned sequences were compared to sequences deposited in the NCBI database (using the Blast search tool). Only PCR sequences with 94–100% matches, and that are pathogens or potential pathogens, are reported here.

### 2.3. Water Turbidity Assessment 

Turbidity measurements were taken using a portable turbidity meter model # 2100P ISO (Hach, Loveland, CO, USA) on site. Turbidity has been used as an indicator of water quality that describes the cloudiness of water caused by suspended particles [23]. Elevated turbidity in an unprotected dug well water may be an indicator of surface water entering the well, or even fine-grained materials entering from the base of the well. In a protected drilled well, elevated turbidity is an indicator of insufficient well development (i.e., removal of fine-grained particles near well screen). 

## 3. Results and Discussion

The average total Heterotrophic Plate Count (HPC) for the open wells (7 × 10^5^ CFU/100 mL) was approximately one order of magnitude higher compared to that of closed wells (9 × 10^4^ CFU/100 mL) and MSABI rope pump wells (7 × 10^4^ CFU/100 mL) (Table 1). There were no statistical differences between closed wells and MSABI rope pump wells (*p* = 0.18). 

As expected, coliforms occur in higher numbers in tropical climates [24,25] and their presence does not always provide a true indication of the presence of human or animal waste in water. All samples examined using the Colilert test kit method were positive for TC with an average of >160 MPN/100 mL for open wells, 102 MPN/100 mL for closed wells and 88 MPN/100 mL for MSABI rope pump wells. However, MSABI rope pump wells and closed wells were within each other’s standard deviation with a *p*-value of 0.20, indicating that there is a twenty percent chance that the results happened accidentally, and therefore are not significant (Table 1). Open wells had higher TC concentration (>160 MPN/100 mL) compared to those of closed wells and MSABI rope pump wells. Water turbidity was higher in open wells, as expected, since open wells are exposed to the environment. Based on the WHO guidelines regarding water turbidity for household water treatment and storage, NTU should be <5 but ideally should be <1 [23]. Even though this guideline does not apply to groundwater, more than half of MSABI rope pump wells had turbidity levels below 5 NTU, while the average was 11 NTU. Closed wells had an average of 28 NTU and that for open wells was 49 NTU (Table 1). 

*E. coli* is a fecal coliform and an indicator of fecal contamination from warm-blooded animals. All water directly intended for drinking should not contain *E. coli* (or thermotolerant bacteria) in any 100-mL sample [14]. *E. coli* was detected in all open wells at concentrations >154 MPN/100 mL. Only four closed wells out of eleven, and two MSABI rope pump wells out of ten, tested positive for *E. coli* with an average concentration of <22 MPN/100 mL (Table 1). While most of the environments surrounding the wells were kept clean, a lack of wellhead protection increases the risks of well contamination from domestic animals and from rainfall.

Of the 93 colonies selected from the 10 wells for species identification by PCR sequencing, 12 were potential pathogens, eight of which came from the open wells: *Enterococcus* spp., *Enterococcus faecalis*, *Clostridium* spp., *Shigella* spp., *Kocuria marina strain* KMM 3905, *Acinetobacter haemolyticus*, *Sphingobacterium composti* and *Bacillus* spp. [26,27,28,29,30,31,32,33,34] (Table 2). *K. marina* and *E. canintestinii* were also found in closed wells, in addition to *Cellulomonas hominis* (or *Oerskovia enterophila*), *Rhodococcus corynebacterioides* DSM 20151, and *Microbacterium* spp. Only one potential pathogen, *Gordonia polyisoprenivorans* [35,36] was found in a MSABI rope pump well.

This study provides a snapshot of the water quality for these water sources. Based on microbial water quality standards and water turbidity (Table 1), the data collected for MSABI rope pump wells and closed wells had comparable microbial water quality. All the measurements for the closed wells and those of MSABI rope pump wells have standard deviations or errors that overlap, indicating that there is no statistical difference. MSABI rope pump wells are 20–30 m in depth and are lined with PVC pipes, compared to closed wells which are >5 m deep. Overall, depth and the type of casing did not appear to be significant factors for better water quality. Providing well lining and cover to prevent outside contamination seems to provide water of good quality. For the intended purposes of diversity, (considering samples that were collected and processed outside the 24-h range provided an additional layer of assessment and a better understanding of the well conditions. Potential pathogens were detected in two of the closed wells and only one of the MSABI rope pump wells. It is important to also note that only a small sample (10 out of the 97) was assessed for microbial diversity. As expected, the open wells performed poorly in all the assessed parameters. Since samples were collected and shipped from Tanzania to the United States, it is expected that some microorganisms may have died and lysed, making it impossible to recover some of them. Therefore, the lack of their recovery does not prove that other microorganisms were not present. 

## 4. Conclusions

The objective of this study was to compare the water quality of three different well designs, in order to help guide future efforts in providing affordable and sustainable interventions to improve access to clean and safe water in rural communities. Previously, MSABI had started to convert open wells into closed wells. However, that program was discontinued before this study began. MSABI rope pump wells were assumed to provide far better water quality than the closed wells (open wells converted into closed wells). However, these initial findings suggest that there may not be a significant difference, given that water sources are protected from outside contamination. In the context of rural areas with limited resources and high poverty rates, interventions that the community themselves can afford are critical in improving access to safe and clean water, as well as fostering ownership in a community. Drilling deeper wells requires drilling machines that need to be brought into the community, which is expensive. As we enter the new phase of achieving SDG goals to improve access to safe and clean water, efforts should be made to explore how the communities themselves can participate in these interventions, to achieve sustainability and ownership. Solutions should coordinate and leverage existing community efforts and explore technologies that are feasible, so as to build sustainability beyond donor funding. 

## Figures and Tables

**Table 1:** Water microbial quality for Maji Safi kwa Afya Bora Ifakara (MSABI) rope pump, closed, and open wells.

	MSABI Rope Pump Wells		Closed Wells		Open Wells			
	Average ± (Stdev)	Samples (n)	Average ± (Stdev)	Samples (n)	Average ± (Stdev)	Samples (n)	*p*-Value ^3^	WHO Guideline (2017)
Total HPC (CFU/100 mL)	7 × 10^4^ ± 3 × 10^2^	30	9 × 10^4^ ± 8 × 10^2^	29	7 × 10^5^ ± 2 × 10^3^	23 (23)	0.18	n.g.v
Total Coliform ^1^ (CFU/100 mL)	2 × 10^3^ ± 1 × 10^3^	34 (23)	3 × 10^3^ ± 3 × 10^3^	30 (18)	4 × 10^4^ ± 3 × 10^4^	29 (29)	0.04	n.g.v
Coliform ^2^ (MPN/100 mL)	>88 ± 29	10 (10)	>102 ± 19	11 (11)	>160	14 (14)	0.20	n.g.v
*E. coli* ^2^ (MPN/100 mL)	<22 ± 6	10 (2)	<22 ± 6	4 (11)	>154 ± 10	14 (14)	n/a	no given value ^4^
Turbidity (NTU)	11 ± 2	34	28 ± 13	31	49 ± 15	29	n/a	<5 ^5^

^1^ detection of total coliform using 3 M Petrifilm Coliform Count Plates; ^2^ detection of total coliform using IDEXX Colilert test kit, ^3^ *p*-value was calculated for MSABI rope pump wells and closed wells only, ^4^ *E. coli* must not be detected in all water directly intended for drinking, ^5^ ideally 1 NTU. However, this may be difficult in certain settings. WHO recommends water disinfection even at 1 NTU (WHO, 2017). The difference between the MSABI rope pump and open wells and closed and that of open wells is a factor of 10 and therefore *p*-value was not calculated, n.g.v: no guideline value, n: number of positives samples, stdev: standard deviation, *p*-value is the estimate of the probability that the results occurred by statistical accident (i.e., *p* < 0.001 means there is 0.1 percent chance the result was an accident), n/a: not applicable.

**Table 2:** Sequence results for cultured bacteria identified as pathogens or potential pathogens.

Water Source	Well ID	Growth Condition	Sequence ID	Blast Accession	Sequence Length	Closest Relative (% Maximum Identity)
Open well	03	APW enrichment/TCBS	Ta	NR_042386.1, or NR_041706.1, or NR_041704.1	1509–1511	*E. canintestinii* strain LMG 13590, *E. sulfureus strain ATCC49903*, *E. casseliflavus* strain (99%)
			Tb	NR_040789.1	1517	*E. faecalis* strain JCM 5803 (98%)
		Blood anaerobic	BNa	NR_041248.1, or NR_024697.1, or NR_036880.1, or NR_043403.1	1306	*B. anthracis* strain ATCC 14578, *B. weihenstephanensis* strain DSM 11821, *B. mycoides* strain 273, *B. thuringiensis* strain IAM 12077 (99%)
			BNb	NR_029249.1, or NR_027573.1, or NR_028611.1	1393–1489	*C. irregulare* strain 6V1, or *C. bartlettii* DSM 13275, *C. hiranonis* DSM 13275 strain TO-931 (99%)
			BNe	NR_027549.1, or NR_02569.1, or NR_026331.1, or NR_02332.1	1473–1494	*E. fergusonii* strain ATCC 35469 or *E. albertinii* or *S. flexneri* strain ATCC 29903 or *S. dysenteriae* strain ATCC 13313 (100%)
	04	APW enrichment/TCBS	Tb	NR_040789.1	1517	*E. faecalis* strain JCM 5803 (99%)
		Blood anaerobic	BNa	NR_042386.1, or NR_041706.1, or NR_041704.1	1498–1511	*E. canintestinii* strain LMG 13590, *E. sulfureus* strain ATCC49903, *E. casseliflavus* strain (99%)
		LB	Le	NR_025723.1	1440	*K. marina strain* KMM 3905 (95%)
	07	APW enrichment/TCBS	Tc	NR_026207.1	1460	*A. haemolyticus* (99%)
			Td	NR_040789.1	1517	*E. faecalis* strain JCM 5803 (98%)
		Blood anaerobic	BNa	NR_041363.1	1433	*S. composti* Ten et al. 2007 strain T5-12 (98%)
Closed well	01	Blood anaerobic	BNa	NR_029288.1, or NR_026239.1	1394–1451	*C. hominis* strain CE40, *O. enterophila* DSM 43856 (100%)
		LB	Lc	NR_025723.1, or NR_027193.1, or NR_026452.1	1440–1481	*K. marina* KMM 3905, *K. carphila* strain CCM 132, *K. rhizophila* strain TA68 (94%)
	07	APW enrichment/TCBS	Tb	NR_042386.1, or NR_041706.1, or NR_041704.1	1498–1511	*E. canintestinii* strain LMG 13590, *E. sulfureus* strain ATCC49903, *E. casseliflavus* strain (99%)
		Blood aerobic	Ba	NR_042262.1, or NR_025548.1	1490	*M. oleivorans* (99%)
		R2A	Rd	NR_041873.1	1494	*R. corynebacterioides* DSM 20151 (99%)
			Rc	NR_037048.1, or NR_042983.1, or NR_026163.1, or NR_026161.1	1429–1526	*M.kitamiense* strain kitami C2, *M. natoiense* strain TNJL143-2, *M. testacum* DSM 20166, *M. imperial* DSM 20530 (100%)
MSABI rope pump well	20	Blood aerobic	Bb	NR_026500.1	1547	*G. polyisoprenivorans* (100%)
Other rope pump well	n/a	LB	Lg	NR_042280.1, or NR_043238.1	1397–1482	*M. llatzerense*, *M. aubagnese* (96%)
			Lf	NR_04988.1, or NR_037031.1, or NR_037030.1	1399–1471	*G. otitidis* strain IFM 10032, *G. sputi* strain 3884, *G. aichiensis* strain E9028 (98%)

APW: Alkaline Peptone Water, LB: Luria-Bertani, TCBS: Thiosulfate Citrate Bile Salts, n/a: not applicable (well design unknown).
